# Peripheral Nerve Conduction Abnormalities and Their Association with Clinical Outcomes in Patients with Unilateral Hemiplegia Secondary to Stroke

**DOI:** 10.3390/jcm15207966

**Published:** 2026-10-15

**Authors:** Selen Özgözen, Pınar Doruk Analan

**Affiliations:** Department of Physical Medicine and Rehabilitation, Dr. Turgut Noyan Training and Research Center, Faculty of Medicine, Baskent University, 01250 Adana, Turkey; doruk.pinar@gmail.com

**Keywords:** stroke rehabilitation, hemiplegia, nerve conduction studies, polyneuropathies, median neuropathy

## Abstract

**Background/Objectives**: Peripheral nerve conduction abnormalities may occur after stroke, but their clinical relevance in chronic hemiplegia remains unclear. This study aimed to characterize these abnormalities and examine their associations with motor and functional outcomes. **Methods**: This single-center, cross-sectional observational study included 72 patients with unilateral hemiplegia ≥ 6 months after ischemic or hemorrhagic stroke. Bilateral lower-extremity nerve conduction studies (NCS) were performed in all participants. In participants without findings suggestive of distal symmetric polyneuropathy (DSP), bilateral upper-extremity NCS were also performed to assess focal entrapment neuropathies; when DSP was suspected from lower-extremity findings, one upper extremity was examined to confirm upper-extremity involvement. Clinical assessments included the Fugl-Meyer Assessment, Functional Independence Measure, Timed Up and Go test, and computerized posturographic fall-risk assessment. Associations between NCS findings and clinical outcomes were examined using adjusted regression analyses. **Results**: Mean age was 61.75 ± 12.56 years, and stroke duration was 42.85 ± 38.14 months. Normal NCS findings were present in 18 (25.0%) patients. Most participants (86.1%) had high posturographic fall-risk. DSP was identified in 38 participants (52.8%). The most frequent focal neuropathy was median nerve entrapment at the wrist (17/72, 23.6%). DSP was more frequent in participants with diabetes than in those without diabetes (85.7% vs. 31.8%; *p* < 0.001). Ulnar sensory nerve action potential amplitude was lower on the paretic side (adjusted *p* = 0.028). Neither DSP nor median nerve entrapment was significantly associated with motor function, functional mobility, or functional independence in adjusted analyses; posturographic fall-risk classifications also did not differ according to these NCS findings in unadjusted exact tests. **Conclusions**: Peripheral nerve conduction abnormalities, particularly DSP and median nerve entrapment, were common in ambulatory individuals with chronic post-stroke hemiplegia. Despite their frequency, no statistically significant associations with motor or functional performance were identified, although clinically meaningful associations cannot be excluded.

## 1. Introduction

Stroke may result in long-term neurological, motor, and functional consequences [1]. As survival after stroke has improved [2], more individuals live for years with persistent disability [3]. In this context, concomitant peripheral nerve disorders may become increasingly relevant to long-term rehabilitation and may add to motor and functional limitations in stroke survivors.

Although stroke results from an acute vascular injury to the central nervous system, its neurological consequences may extend beyond the central lesion. Peripheral electrophysiological alterations have been reported after stroke, although their underlying mechanisms remain incompletely understood [4,5,6,7].

Nerve conduction studies (NCS) have demonstrated peripheral abnormalities after stroke [7,8,9,10]. However, studies have differed substantially in stroke chronicity, motor severity, nerves examined, electrophysiological methodology, and clinical outcomes assessed [4,5,11,12].

The clinical relevance of these peripheral electrophysiological abnormalities, particularly in individuals with chronic motor impairment, remains uncertain. Therefore, we aimed to characterize peripheral nerve conduction abnormalities across multiple upper- and lower-extremity nerves in ambulatory individuals with chronic hemiplegia after stroke and to investigate their associations with motor and functional outcomes.

## 2. Materials and Methods

This single-center, cross-sectional observational study with prospective data collection was conducted at the Department of Physical Medicine and Rehabilitation of Başkent University Faculty of Medicine, from October 2024 to June 2026. It evaluated the frequency of peripheral nerve conduction abnormalities and their associations with motor and functional outcomes in adults with unilateral hemiplegia after ischemic or hemorrhagic stroke. The protocol was approved by the institutional review board (KA24/315), written informed consent was obtained from all participants, and the study was registered at ClinicalTrials.gov (NCT07685691, initial release: 29 June 2026).

### 2.1. Participants

**Figure 1:**
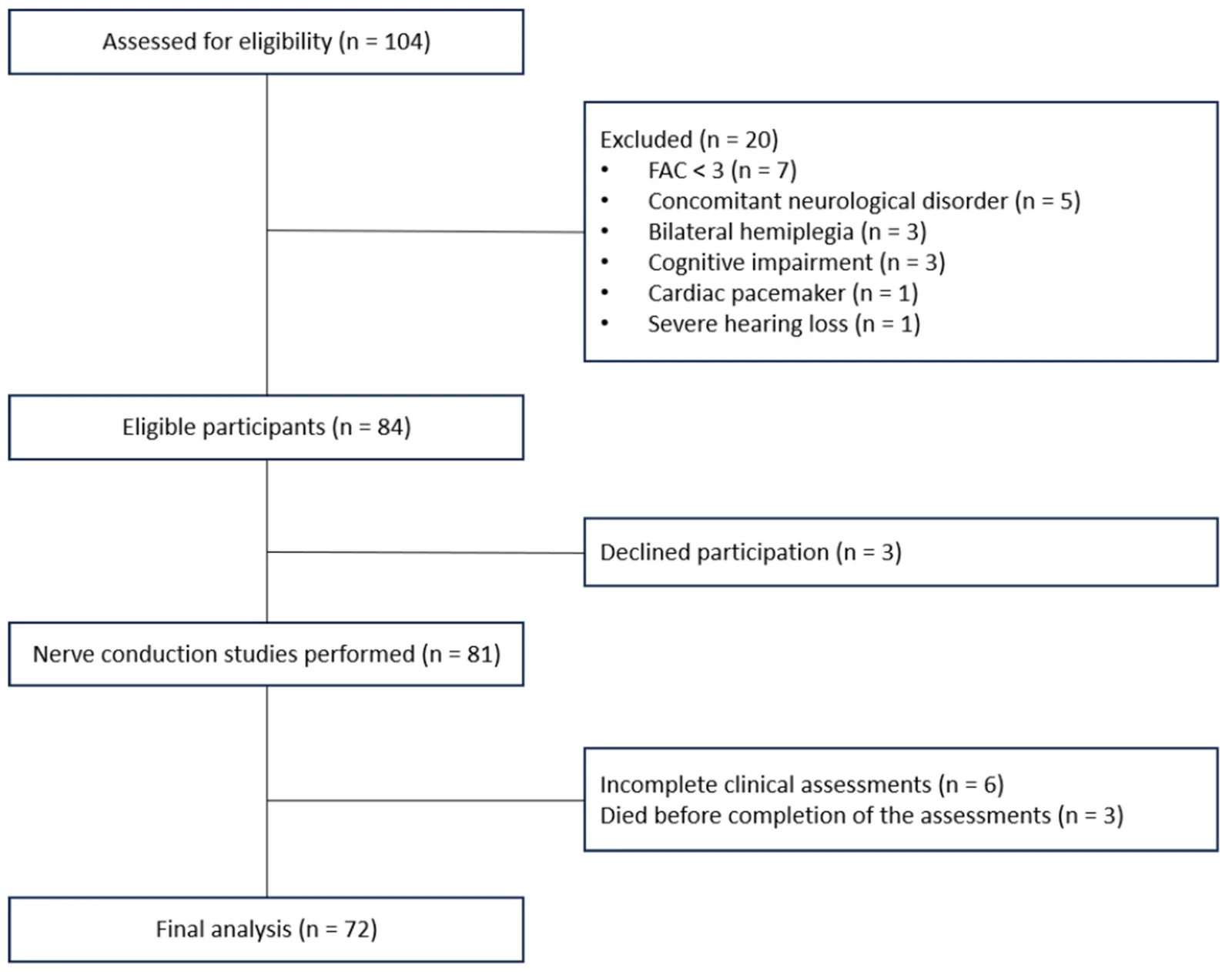
Flow diagram of the study population.

Adults (≥18 years) at least 6 months after stroke were consecutively screened. Inclusion required unilateral hemiplegia, Functional Ambulation Category (FAC) ≥ 3, ability to complete the 3 m Timed Up and Go (TUG) test and computerized static posturography, and informed consent. Exclusion criteria were bilateral hemiplegia; hemiplegia due to intracranial tumor; fracture, amputation, skin lesions precluding NCS, or diagnosed peripheral vascular disease; neurological disorders affecting motor function or peripheral nerve conduction; conditions precluding safe NCS; or inability to cooperate with examinations. The study flow is presented in Figure 1.

Brain lesions were classified from neuroimaging as cortical/cortico-subcortical, pure subcortical, infratentorial, or mixed, adapted from Chu et al. [13]. Pure subcortical lesions involved deep gray or white matter without cortical involvement; brainstem/cerebellar lesions were infratentorial.

### 2.2. Clinical Assessments

Age, sex, stroke etiology and duration, lesion localization, and comorbidities were recorded. The same experienced physician performed all assessments. Motor function was evaluated with the Fugl-Meyer Assessment–Upper Extremity (FMA-UE; 0–66) and Fugl-Meyer Assessment–Lower Extremity (FMA-LE; 0–34) [14], functional independence with the Functional Independence Measure (FIM; 18–126) [15], and mobility with 3 m TUG [16]. Higher FMA and FIM scores indicated better function, whereas longer TUG times indicated poorer mobility.

Computerized static posturography was performed barefoot using Tetrax^®^ (Sunlight Medical Ltd., 3 Weizmann Science Park, Rehovot 76100, Israel) in eight standardized 32 s standing conditions according to the manufacturer’s protocol. The Fall Index was analyzed and categorized as low (0–36), moderate (37–58), or high (59–100) risk [17]. The same experienced physician performed all assessments. Assessors were not formally blinded to the results of the other study assessments. The study protocol specified a target assessment period of 30 days, and NCS and clinical assessments were scheduled as close together as practically feasible. Exact patient-level intervals between NCS and clinical assessments were not systematically recorded.

### 2.3. Nerve Conduction Studies

All NCS were performed using a standardized protocol under the direct supervision of the same experienced electrodiagnostic physician with a Medelec Synergy four-channel electromyography system (Oxford Instruments Medical Ltd., Manor Way, Old Woking, Surrey GU22 9JU, UK). Motor responses were recorded using 9 mm Ag/AgCl surface disc electrodes (TECA Accessories, Medelec/Oxford Instruments Medical Ltd., Manor Way, Old Woking, Surrey GU22 9JU, UK) over the motor points of the abductor pollicis brevis, abductor digiti minimi, extensor digitorum brevis, and abductor hallucis for the median, ulnar, peroneal, and tibial nerves, respectively. Median and ulnar sensory nerve action potentials (SNAP) were recorded antidromically with ring electrodes from digits III and V, respectively; sural and superficial peroneal sensory responses were also recorded antidromically using surface disc electrodes.

Motor and F-wave studies used supramaximal stimulation. Motor distal latency was measured to compound muscle action potential (CMAP) onset and CMAP amplitude from baseline to the first negative peak; sensory latency was measured to the SNAP peak and SNAP amplitude peak-to-peak. Motor and sensory conduction velocities were expressed in m/s. For F-wave assessment, 6–10 stimuli were delivered and minimum F-wave latency was recorded. Filter settings were 3 Hz–10 kHz for motor and 20 Hz–2 kHz for sensory studies. Standard stimulation sites were the wrist and antecubital fossa for the median nerve, wrist and below/above elbow for the ulnar nerve, ankle and popliteal fossa for the tibial nerve, and ankle and below/above fibular head for the peroneal nerve. Median palm-to-wrist mixed NCS were performed by stimulating the nerve in the palm and recording at the wrist using surface disc electrodes. Testing was performed in a temperature-controlled room, and cool limbs were warmed when necessary.

Nerve conduction parameters were considered abnormal when distal or F-wave latencies exceeded the established upper reference limits, or when CMAP/SNAP amplitudes and motor or sensory conduction velocities fell below the established lower reference limits [18]. The same laboratory reference limits were used throughout. For motor studies, the reference limits for the median, ulnar, peroneal, and tibial nerves were as follows, respectively: distal latency, 4.00/3.45/5.23/5.23 ms; CMAP amplitude, 4.25/4.32/2.16/5.56 mV; conduction velocity, 50.3/53.6/43.13/41.68 m/s; and minimum F-wave latency, 27.87/28.41/53.06/53.51 ms.

For sensory studies, the reference limits were: median nerve, latency 3.41 ms, SNAP amplitude 10 μV, and conduction velocity 50.6 m/s; ulnar nerve, latency 3.41 ms, SNAP amplitude 11 μV, and conduction velocity 54.2 m/s; sural nerve, SNAP amplitude 9.86 μV and conduction velocity 34.08 m/s; and superficial peroneal nerve, SNAP amplitude 4 μV, and conduction velocity 40.5 m/s. For median mixed nerve studies, the lower reference limits were 32.4 μV for amplitude and 35.9 m/s for conduction velocity.

NCS were initiated in all participants with bilateral tibial and peroneal motor studies, followed by bilateral sural and superficial peroneal sensory studies. When lower-extremity findings suggested DSP, one upper extremity was subsequently examined to determine whether the abnormalities extended to the upper limb; the side examined was not prespecified. In these participants, the purpose of upper-extremity testing was confirmation of a generalized neuropathic pattern rather than systematic bilateral assessment for focal entrapment neuropathy. In participants without findings suggestive of DSP, bilateral upper-extremity NCS were subsequently performed to assess focal entrapment neuropathies. In one participant with DSP, bilateral upper-extremity NCS were performed based on clinical findings, as an exception to the standard examination protocol. For the purposes of this study, DSP was classified electrophysiologically and required abnormalities in at least two separate nerves, including the sural nerve, according to AAN/AANEM/AAPM&R criteria [19]. Focal entrapment neuropathies were identified by abnormalities localized to the characteristic entrapment segment and not attributable to a generalized neuropathic process. Median nerve entrapment at the wrist was defined by focal slowing of median nerve conduction across the wrist, reflected by prolonged distal motor and/or sensory latency or abnormal palm-to-wrist mixed nerve conduction, with or without reduced median CMAP or SNAP amplitude. Ulnar nerve entrapment at the elbow was defined by motor conduction velocity below the laboratory reference limit (53.6 m/s) across the elbow segment, together with slowing relative to the adjacent forearm segment. Peroneal nerve entrapment at the fibular head was defined by motor conduction velocity below the laboratory reference limit (43.13 m/s) across the fibular head segment, together with slowing relative to the adjacent lower-leg segment. No additional numerical cutoff for intersegmental slowing was applied. Absent responses were considered abnormal for diagnostic classification and were excluded from statistical analyses of continuous nerve conduction parameters. Normal NCS was defined as normal findings across all nerve conduction parameters, with all measured values within the laboratory reference limits.

### 2.4. Statistical Analysis

An a priori power analysis was performed by the institutional Statistical Consulting and Research Center using G*Power 3.1.9.4. An effect size of d = 0.4375 was selected based on a previous electrophysiological study of entrapment neuropathies in patients with chronic stroke [20]. With a two-sided α = 0.05 and 95% power, a one-sample t-test calculation yielded a minimum sample size of 70 participants. This calculation was used to determine the overall recruitment target and was not specifically designed to power the subsequent multivariable regression analyses. In response to the limited sample size relative to the multivariable analyses, post hoc power calculations were additionally performed using G*Power 3.1.9.7 and fixed-effects multiple linear regression models based on the observed R^2^ values. With an analytical sample of 69 participants, eight predictor degrees of freedom, and α = 0.05, achieved power across the eight adjusted models ranged from 57.8% to 88.6%. These calculations reflect the power of the overall regression models rather than that of the individual NCS predictor coefficients and indicate limited power for some of the adjusted analyses.

Analyses were performed using IBM SPSS Statistics for Windows, version 26.0. Normality was assessed by the Shapiro–Wilk test and visual inspection of histograms and Q–Q plots. Data are presented as mean ± SD, median (Q1–Q3), or n (%), as appropriate.

Correlations among clinical outcomes were evaluated using Spearman’s rank correlation coefficient. Paretic/non-paretic NCS parameters were compared using paired-samples t tests for normally distributed variables and Wilcoxon signed-rank tests for non-normally distributed variables. These side-to-side comparisons were considered exploratory, and *p* values for the 28 comparisons were adjusted for multiple testing using the Holm–Bonferroni method. Continuous NCS analyses included evaluable responses only; absent responses and measurements not performed according to protocol were excluded from the corresponding analyses. The association between diabetes mellitus and DSP was evaluated using the Pearson chi-square test.

Multiple linear regression analyses assessed independent associations of DSP and any median entrapment neuropathy with FMA-UE, FMA-LE, FIM, and TUG, adjusting for age, sex, stroke duration, diabetes mellitus, and lesion localization. Lesion localization was entered as a categorical variable with cortical/cortico-subcortical lesions as the reference. Participants with missing lesion-localization data (n = 3) were excluded from the adjusted regression models by listwise deletion. TUG was natural log-transformed. Unstandardized B values with 95% CIs are reported. Model assumptions and influential observations were assessed using tolerance, VIF, standardized residuals, Cook’s distance, and leverage, with sensitivity analyses excluding influential observations. Side-specific median entrapment analyses were not performed because the paretic-side, non-paretic-side, and bilateral subgroups were small and upper-extremity examination was not uniform across the entire cohort; in participants with lower-extremity findings suggestive of DSP, only one upper extremity was examined, whereas bilateral upper-extremity NCS were performed in participants without findings suggestive of DSP. Consequently, bilateral assessment for median entrapment was not available in all participants, which limited the comparability and interpretation of the side-specific median entrapment subgroups and, together with the small subgroup sizes, precluded reliable side-specific multivariable analyses. As an exploratory sensitivity analysis, paretic- and non-paretic-side sural SNAP amplitudes were separately examined as continuous electrophysiological predictors using the same adjusted regression models. Sural SNAP amplitude was selected because bilateral sural studies were systematically performed in all participants and because it provides a continuous measure directly relevant to the electrophysiological assessment of DSP. Posturographic fall-risk categories were compared using the Fisher–Freeman–Halton exact test. All tests were two-tailed; *p* < 0.05 was considered statistically significant.

ChatGPT (OpenAI, Plus subscription) was used solely to improve the English language, readability, and clarity of the manuscript. As the model version may have changed during manuscript preparation, a single version could not be reliably specified.

## 3. Results

### 3.1. Characteristics of Participants

Seventy-two patients with unilateral post-stroke hemiplegia were included; mean age was 61.75 ± 12.56 years, 52 (72.2%) were male, and mean stroke duration was 42.85 ± 38.14 months. Ischemic stroke occurred in 55 participants (76.4%) and hemorrhagic stroke in 17 (23.6%). Hypertension and diabetes mellitus were present in 48 (66.7%) and 28 (38.9%), respectively.

Mean FMA-UE, FMA-LE, and FIM scores were 35.61 ± 18.85, 21.71 ± 6.67, and 103.89 ± 11.35, respectively, and median TUG time was 18.35 s (IQR, 13.70–27.00). Most participants (86.1%) were classified as having high posturographic fall risk. Demographic and clinical characteristics are summarized in Table 1.

### 3.2. Correlations of Clinical Outcome Measures

All clinical outcome measures were significantly correlated with each other (all *p* < 0.01). Better upper- and lower-extremity motor function was associated with better mobility and greater functional independence, whereas poorer motor function and mobility were associated with higher Fall Index scores. The strongest association between distinct clinical measures was observed between TUG and FIM (ρ = −0.622). The complete correlation matrix is presented in Table 2.

### 3.3. Nerve Conduction Study Findings

Of the 72 participants, 38 (52.8%) had DSP and 18 (25.0%) had normal NCS findings. DSP was present in 24 of 28 participants with diabetes (85.7%) compared with 14 of 44 without diabetes (31.8%), a significant difference (χ^2^ = 19.943, *p* < 0.001). Median nerve entrapment was identified in 17 participants (23.6%) overall. Ulnar and peroneal entrapments were identified in 2 and 4 participants, respectively, whereas no tibial entrapment neuropathy was detected. Median entrapment was present on the paretic side in 6 participants, on the non-paretic side in 6, and bilaterally in 5. Detailed NCS findings are presented in Table 3.

### 3.4. Comparison of Paretic and Non-Paretic Limbs

Side-to-side comparisons of NCS parameters were considered exploratory. Before adjustment for multiple comparisons, significant side-to-side differences were observed in ulnar motor distal latency, CMAP amplitude, and SNAP amplitude, as well as in tibial and peroneal minimum F-wave latencies. After Holm–Bonferroni correction for the 28 comparisons, only the difference in ulnar SNAP amplitude remained statistically significant, with lower values on the paretic side (14.90 [9.93–23.45] vs. 21.95 [13.00–31.75] µV; Hodges–Lehmann paired difference [non-paretic minus paretic] = 8.95 µV, 95% CI 3.60 to 15.55; adjusted *p* = 0.028). No other side-to-side differences remained statistically significant after correction for multiple testing (Table 4).

### 3.5. Associations Between NCS Abnormalities and Clinical Outcomes

In analyses adjusted for age, sex, stroke duration, diabetes mellitus, and lesion localization, neither DSP nor median entrapment neuropathy was significantly associated with FMA-UE, FMA-LE, FIM, or TUG (Table 5). Posturographic fall-risk categories also did not differ according to the presence of DSP or median entrapment neuropathy (all *p* > 0.05). Given the small numbers of participants with ulnar (n = 2) and peroneal (n = 4) entrapment neuropathies, these findings were reported descriptively and no inferential comparisons were performed. All adjusted regression models included 69 participants. Collinearity diagnostics indicated no evidence of problematic multicollinearity (maximum VIF = 1.608). In the exploratory sensitivity analyses using sural SNAP amplitude as a continuous predictor, sural responses were absent in 15 participants on each side. After excluding absent responses and participants with incomplete covariate data, 55 participants were included in each of the eight regression models (Supplementary Table S1). Neither paretic- nor non-paretic-side sural SNAP amplitude was significantly associated with FMA-UE, FMA-LE, FIM, or ln(TUG) (all *p* > 0.28). Sensitivity analyses excluding the two extreme TUG observations (156 and 230 s; n = 67) did not materially alter the results for DSP (B = −0.055, 95% CI −0.381 to 0.272; *p* = 0.739) or median entrapment neuropathy (B = −0.118, 95% CI −0.434 to 0.198; *p* = 0.459).

## 4. Discussion

In the present study, peripheral nerve conduction abnormalities were common, with only 25.0% of participants showing normal NCS findings. DSP was the most common finding (52.8%), whereas median nerve entrapment was the most frequent focal neuropathy (23.6%). Ulnar and peroneal entrapments were uncommon, and no tibial entrapment neuropathy was identified. The only significant side-to-side difference after adjustment for multiple comparisons was a lower ulnar SNAP amplitude on the paretic side. Clinically, residual motor impairment persisted despite relatively preserved functional independence, and most participants (86.1%) were classified as having high posturographic fall-risk. Neither DSP nor median nerve entrapment was significantly associated with motor or functional outcomes. Although no statistically significant associations of peripheral nerve conduction abnormalities with motor or functional outcomes were identified, the wide confidence intervals around the regression estimates do not exclude clinically meaningful associations.

As expected, our clinical outcome measures showed a coherent pattern of associations. Better upper- and lower-extremity motor function was associated with greater functional independence and better mobility, whereas poorer motor function and mobility were associated with higher fall risk. The strongest association among distinct clinical measures was between TUG and FIM, supporting a consistent clinical profile across complementary measures of motor and functional status.

Comparison with previous studies is complicated by substantial methodological heterogeneity, including differences in stroke chronicity, ambulatory status, motor severity, nerves examined, electrophysiological methodology, and study populations [4,5,11,12]. Some studies also excluded diabetes, polyneuropathy, or entrapment neuropathies [7], whereas these conditions were among the principal findings of our study. These differences should be considered when comparing reported frequencies and clinical associations.

The high frequency of DSP in our cohort is consistent with previous reports of generalized peripheral nerve abnormalities after stroke. Peripheral polyneuropathy was reported in 42% of 30 post-stroke patients assessed at a mean of 5.9 months after stroke [9]. However, the electrophysiological criteria used to define polyneuropathy were not clearly specified, limiting direct comparison with our DSP classification. Another study examined the non-paretic limbs of patients with ischemic stroke while excluding diabetes, impaired glucose tolerance, and known peripheral nerve disease. The non-paretic limb was deliberately examined to minimize secondary effects related to paresis. Compared with age-matched controls, stroke patients more frequently demonstrated abnormalities in ulnar and common peroneal conduction parameters, suggesting a broader susceptibility to peripheral neuropathies [7]. In our cohort, DSP was substantially more frequent among participants with diabetes than among those without diabetes. Given the common vascular and metabolic comorbidities in our cohort and the absence of an age- and comorbidity-matched healthy control group, the 52.8% DSP frequency should be interpreted cautiously and viewed primarily as a characteristic of this chronic stroke cohort.

Focal entrapment neuropathies have also been reported after stroke. NCS performed during the first days after stroke and again 45–50 days later demonstrated frequent CTS, cubital tunnel syndrome, and axonal abnormalities in severely paretic limbs, whereas CTS was also detected in the non-paretic upper extremity of more mobile patients, particularly those using assistive devices [8]. Median, ulnar, and peroneal entrapment neuropathies have similarly been reported, particularly in severely paretic limbs [21]. In our cohort, however, no clear predominance was observed among paretic, non-paretic, and bilateral median entrapment neuropathies.

Previous studies have reported variable paretic–non-paretic differences in NCS parameters. Lower median and ulnar CMAP amplitudes, prolonged tibial distal motor latency and median F-wave latency, and slower tibial motor conduction velocity have been reported on the paretic side [10]. Lower median and ulnar CMAP amplitudes without significant differences in conduction velocities have also been described [22], whereas another study found lower M-response amplitudes and prolonged F-wave latencies despite preserved distal motor latencies and conduction velocities [6]. In contrast, no significant paretic–non-paretic differences in common peroneal CMAP amplitude or motor conduction velocity were found during the acute phase [12]. In our cohort, after adjustment for multiple comparisons, the only significant interlimb difference was a lower ulnar SNAP amplitude on the paretic side. Thus, our findings indicate relatively limited side-to-side electrophysiological differences in this chronic stroke cohort. Stroke chronicity may partly contribute to this variability. Mean post-stroke duration was approximately 14 months in one previous cohort [10] and 43 months in ours. Positive correlations between stroke duration and paretic-side median and ulnar CMAP amplitudes have also been reported, suggesting that electrophysiological recovery may occur over time and potentially contribute to differing patterns in longer-term cohorts [10]. Beyond conventional NCS parameters, motor-unit, needle electromyography, and axonal excitability studies have demonstrated peripheral changes after stroke, without indicating a single uniform electrophysiological pattern [4,5,11,23].

Associations between peripheral electrophysiological findings and clinical function have also varied across studies. Peripheral neuropathies have been suggested to adversely affect walking and increase fall risk after stroke, although direct associations between NCS abnormalities and functional outcomes were not tested [7]. In our study, these associations were directly examined, and DSP and focal entrapment neuropathies were not significantly related to motor function, mobility, functional independence, or posturographic fall-risk classification. Higher paretic-side median and ulnar CMAP amplitudes have been associated with better motor recovery, motor FIM scores, and ambulatory function [10]. This difference may reflect the analytical approaches used: the previous study evaluated individual nerve conduction parameters, particularly CMAP amplitudes, whereas our primary analyses focused on electrophysiologically defined abnormalities such as DSP and focal entrapment neuropathy. In exploratory sensitivity analyses using sural SNAP amplitude as a continuous electrophysiological measure, neither paretic- nor non-paretic-side amplitudes were significantly associated with the motor or functional outcomes, although the reduced analyzable sample limits interpretation of these findings. No significant relationship has also been found between changes in paretic-side common peroneal CMAP amplitude or motor conduction velocity and improvement in lower-limb motor strength [12], while another study similarly reported no significant relationship between clinical and electroneurographic findings [22]. Taken together, these observations are consistent with our finding that peripheral electrophysiological abnormalities did not show a clear relationship with current motor and functional performance. Nevertheless, our cohort included only ambulatory patients with FAC ≥ 3, which may have narrowed the range of disability and reduced the ability to detect associations more evident in severely impaired or non-ambulatory patients.

### Study Limitations

Several limitations should be considered. The absence of an age- and comorbidity-matched control group prevents determining whether the observed frequency of DSP or focal neuropathies exceeds that expected in a comparable population. Upper-extremity NCS were not performed bilaterally in all participants. In patients evaluated with the unilateral upper-extremity polyneuropathy protocol, focal median or ulnar entrapment neuropathy on the unexamined side may therefore have been missed, potentially leading to underestimation of the overall frequency and limiting interpretation of side-specific entrapment distributions. Inclusion of only ambulatory patients with chronic hemiplegia limits generalizability to acute, severely affected, or non-ambulatory populations and may have narrowed the range of functional outcomes. Although examinations were performed in a temperature-controlled room and extremities were warmed when necessary, skin temperature was not quantitatively measured or recorded; therefore, a contribution of interlimb temperature differences to side-to-side NCS findings cannot be completely excluded. Spasticity was not systematically quantified and therefore could not be examined in relation to the electrophysiological findings. Spasticity was not a primary construct of interest; motor impairment and functional performance were characterized using motor and functional outcome measures. Small ulnar and peroneal entrapment subgroups limited subgroup analyses. In addition, the achieved power of some adjusted regression models was limited, reducing the ability to detect modest associations. Dedicated neuropathic pain questionnaires and detailed peripheral sensory assessments were not included, although central sensory deficits after stroke may complicate such assessments. Potential causes of peripheral neuropathy other than diabetes, including chronic alcohol consumption, autoimmune disorders, nutritional deficiencies, thyroid dysfunction, and chemotherapy history, were not systematically collected; therefore, residual confounding by these factors cannot be excluded. Finally, pre-stroke limb dominance was not incorporated into the analyses.

Despite these limitations, the study provides NCS data from multiple upper- and lower-extremity nerves within the same chronic ambulatory stroke cohort and evaluates both generalized and focal peripheral nerve abnormalities in relation to several complementary motor and functional outcomes. By including patients with common vascular and metabolic comorbidities, the study reflects the clinical heterogeneity encountered in routine chronic stroke practice.

## 5. Conclusions

In conclusion, both DSP and median nerve entrapment appear to commonly coexist in patients with chronic hemiplegia after stroke. However, no statistically significant associations between peripheral nerve abnormalities and motor or functional performance were identified in this ambulatory cohort; the wide confidence intervals do not exclude clinically meaningful associations.

## Figures and Tables

**Table 1:** Demographic and clinical characteristics of the study population (n = 72).

Variable	
Age (years)	61.75 ± 12.56
Sex, male, n (%)	52 (72.2)
Stroke duration (months)	42.85 ± 38.14
Ischemic stroke, n (%)	55 (76.4)
Hemorrhagic stroke, n (%)	17 (23.6)
Side of hemiplegia, n (%)	
Right	40 (55.6)
Left	32 (44.4)
Stroke lesion location, n (%)	
Cortical/cortico-subcortical	24 (34.8)
Pure subcortical	23 (33.3)
Infratentorial	12 (17.4)
Mixed	10 (14.5)
Imaging unavailable	3 (4.2)
Hypertension, n (%)	48 (66.7)
Diabetes mellitus, n (%)	28 (38.9)
FMA-UE (0–66)	35.61 ± 18.85
FMA-LE (0–34)	21.71 ± 6.67
FIM (18–126)	103.89 ± 11.35
TUG (s)	18.35 (13.70–27.00)
Posturographic fall-risk classification, n (%)	
Low risk (0–36)	6 (8.3)
Moderate risk (37–58)	4 (5.6)
High risk (59–100)	62 (86.1)

Data are presented as mean ± SD, median (Q1–Q3), or n (%), as appropriate. Percentages for lesion-location categories were calculated among participants with available neuroimaging data (n = 69); imaging unavailable was calculated based on the total study population (n = 72). FMA, Fugl-Meyer Assessment; UE, Upper Extremity; LE, Lower Extremity; FIM, Functional Independence Measure; TUG, Timed Up and Go.

**Table 2:** Correlation analysis of clinical outcome measures in the study population (n = 72).

Variable	FMA-UE	FMA-LE	TUG	Fall Index	FIM
FMA-UE	—	0.623	−0.405	−0.383	0.508
FMA-LE		—	−0.543	−0.324	0.492
TUG			—	0.348	−0.622
Fall Index				—	−0.347
FIM					—

Values are Spearman’s correlation coefficients (ρ). All correlations were statistically significant at *p* < 0.01 (two-tailed). FIM, Functional Independence Measure; FMA-UE, Fugl-Meyer Assessment–Upper Extremity; FMA-LE, Fugl-Meyer Assessment–Lower Extremity; TUG, Timed Up and Go.

**Table 3:** Distribution of nerve conduction study findings (n = 72).

Nerve Conduction Study Finding	n (%)
Distal symmetric polyneuropathy Without concomitant focal entrapment With concomitant median entrapment neuropathy	38 (52.8) 36 (50.0) 2 (2.8)
Normal NCS	18 (25.0)
Isolated median entrapment neuropathy	12 (16.7)
Combined focal entrapment neuropathies * Paretic-side median + paretic-side peroneal neuropathies Non-paretic-side median + paretic-side peroneal neuropathies Non-paretic-side ulnar + paretic-side peroneal neuropathies Bilateral median + non-paretic-side ulnar + paretic-side peroneal neuropathies	4 (5.6) 1 (1.4) 1 (1.4) 1 (1.4) 1 (1.4)

Percentages are calculated based on the total study population. * Combined focal entrapment neuropathies included combinations of median, ulnar, and/or peroneal entrapment neuropathies without distal symmetric polyneuropathy. NCS, Nerve Conduction Study.

**Table 4:** Comparison of nerve conduction study parameters between the paretic and non-paretic sides.

Nerve	Paretic Side	Non-Paretic Side	Paired n	Adjusted *p*
**Median nerve**				
Motor distal latency (ms)	3.70 (3.25–4.06)	3.80 (3.50–4.35)	34	1.000
CMAP amplitude (mV)	7.41 ± 2.52	7.47 ± 2.77	34	1.000
Motor CV (m/s)	54.47 ± 5.45	56.37 ± 4.47	34	1.000
Minimum F-wave latency (ms)	26.43 (25.05–28.55)	26.60 (25.25–28.70)	35	1.000
Sensory distal latency (ms)	3.15 (2.85–3.54)	3.33 (3.05–3.90)	35	1.000
SNAP amplitude (µV)	28.14 ± 14.20	31.36 ± 13.15	35	1.000
Sensory CV (m/s)	54.75 (51.70–59.45)	54.30 (49.90–58.70)	34	1.000
Palm-to-wrist mixed amplitude (µV)	40.20 (32.65–47.50)	37.00 (33.05–44.10)	33	1.000
Palm-to-wrist mixed CV (m/s)	39.30 (34.50–40.50)	37.45 (31.55–41.35)	33	1.000
Absent responses, n (%)	1 (1.4%)	1 (1.4%)		
**Ulnar nerve**				
Motor distal latency (ms)	2.98 ± 0.39	2.79 ± 0.27	34	0.162
CMAP amplitude (mV)	8.49 ± 2.08	9.17 ± 1.84	34	0.754
Motor CV (m/s)	64.38 ± 7.46	63.11 ± 7.75	34	1.000
Minimum F-wave latency (ms)	26.25 ± 2.01	25.79 ± 1.97	34	1.000
Sensory distal latency (ms)	3.00 (2.73–3.15)	3.03 (2.75–3.25)	35	1.000
SNAP amplitude (µV)	14.90 (9.93–23.45)	21.95 (13.00–31.75)	35	**0** **.028**
Sensory CV (m/s)	57.95 (54.55–65.45)	56.80 (51.80–62.43)	35	1.000
**Tibial nerve**				
Motor distal latency (ms)	3.90 (3.50–4.95)	3.90 (3.63–4.65)	67	1.000
CMAP amplitude (mV)	7.50 (5.60–10.30)	7.40 (5.60–10.85)	67	1.000
Motor CV (m/s)	43.40 ± 6.56	43.92 ± 5.69	67	1.000
Minimum F-wave latency (ms)	48.90 (46.25–54.35)	49.00 (45.75–53.45)	67	0.960
Absent responses, n (%)	5 (6.9%)	3 (4.2%)		
**Peroneal nerve**				
Motor distal latency (ms)	4.23 (3.85–4.60)	4.03 (3.75–4.59)	61	1.000
CMAP amplitude (mV)	3.33 ± 1.61	3.45 ± 1.64	61	1.000
Motor CV (m/s) *	43.75 (39.25–48.98)	44.95 (40.38–48.73)	61	1.000
Minimum F-wave latency (ms)	48.78 (45.11–54.01)	47.38 (44.45–53.38)	61	0.775
Absent responses, n (%)	8 (11.1%)	6 (8.3%)		
**Sural nerve**				
SNAP amplitude (µV)	10.99 ± 5.36	10.73 ± 5.80	55	1.000
Sensory CV (m/s)	38.18 ± 4.78	38.68 ± 4.82	55	1.000
Absent responses, n (%)	15 (20.8%)	15 (20.8%)		
**Superficial peroneal nerve**				
SNAP amplitude (µV)	9.90 (5.30–11.80)	8.80 (4.95–12.40)	45	1.000
Sensory CV (m/s)	39.10 (35.40–41.50)	38.60 (35.95–40.95)	45	1.000
Absent responses, n (%)	25 (34.7%)	23 (31.9%)		

Continuous parameters are presented for evaluable responses only. Paired-samples t-test was used for variables presented as mean ± SD, and the Wilcoxon signed-rank test was used for variables presented as median (Q1–Q3). * Motor conduction velocity for the peroneal nerve was calculated across the ankle-to-below fibular head segment. *p* values for the 28 side-to-side comparisons were adjusted for multiple testing using the Holm–Bonferroni method. CV, conduction velocity; CMAP, compound muscle action potential; SNAP, sensory nerve action potential.

**Table 5:** Associations between NCS abnormalities and clinical outcomes.

A. Main Analyses						
NCS Abnormality	Clinical Outcome	Adjusted B (95% CI)	*p*	R^2^	Adjusted R^2^	Overall F-Test *p*
Distal symmetric polyneuropathy	FMA-UE FMA-LE	0.36 (−10.50 to 11.21) 0.05 (−3.96 to 4.05)	0.948 0.982	0.225 0.170	0.122 0.059	0.041 0.165
	FIM	0.15 (−6.45 to 6.75)	0.964	0.201	0.094	0.079
	TUG	0.040 (−0.356 to 0.436)	0.841	0.139	0.024	0.308
	Posturographic fall risk	—	0.774 *	—	—	—
Any median entrapment neuropathy	FMA-UE	3.70 (−6.87 to 14.26)	0.487	0.232	0.129	0.035
	FMA-LE	1.33 (−2.57 to 5.23)	0.496	0.176	0.066	0.143
	FIM	−2.19 (−8.61 to 4.24)	0.498	0.207	0.101	0.068
	TUG	−0.146 (−0.531 to 0.239)	0.451	0.147	0.033	0.267
	Posturographic fall risk	—	0.270 *	—	—	—

Adjusted linear regression models included 69 participants and were adjusted for age, sex, stroke duration, diabetes mellitus, and lesion localization. B values are unstandardized coefficients with 95% CIs; TUG was natural log-transformed. R^2^, adjusted R^2^, and overall F-test *p* values refer to the adjusted regression models. * Posturographic fall-risk classification was analyzed using the unadjusted Fisher–Freeman–Halton exact test. FIM, Functional Independence Measure; FMA-UE, Fugl-Meyer Assessment–Upper Extremity; FMA-LE, Fugl-Meyer Assessment–Lower Extremity; NCS, Nerve Conduction Study; TUG, Timed Up and Go.

## Data Availability

The data supporting the findings of this study are available from the corresponding author upon reasonable request.

## Supplementary Materials

The following supporting information can be downloaded at: https://www.mdpi.com/article/10.3390/jcm15207966/s1, Supplementary Table S1. Associations of paretic and non-paretic sural SNAP amplitudes with clinical outcomes in adjusted linear regression analyses.
