# Supplementary material for: Peripheral Nerve Conduction Abnormalities and Their Association with Clinical Outcomes in Patients with Unilateral Hemiplegia Secondary to Stroke
Source: J Clin Med. 2026 Oct 15;15(20):7966. doi: 10.3390/jcm15207966 (PMC13705962; doi:10.3390/jcm15207966)
Supplement: Supplementary file 1 [file jcm-15-07966-s001.zip › jcm-4606907-supplementary.pdf]

**Supplementary Table S1. Associations of paretic and non-paretic sural SNAP amplitudes with clinical outcomes in adjusted linear regression analyses.**

| Predictor | Outcome | n | B (95% CI) | p |
|---|---|---|---|---|
| Paretic sural SNAP | FMA-UE | 55 | −0.542 (−1.546 to 0.461) | 0.282 |
| | FMA-LE | 55 | −0.083 (−0.466 to 0.299) | 0.664 |
| | FIM | 55 | −0.172 (−0.851 to 0.507) | 0.613 |
| | ln(TUG) | 55 | 0.007 (−0.027 to 0.042) | 0.661 |
| Non-paretic sural SNAP | FMA-UE | 55 | −0.364 (−1.321 to 0.593) | 0.448 |
| | FMA-LE | 55 | 0.071 (−0.294 to 0.437) | 0.697 |
| | FIM | 55 | 0.180 (−0.459 to 0.819) | 0.573 |
| | ln(TUG) | 55 | 0.001 (−0.036 to 0.039) | 0.947 |

All models were adjusted for age, sex, stroke duration, diabetes mellitus, and lesion localization. Sural SNAP responses were absent in 15 participants on each side; 55 participants were included in each model after exclusion of absent responses and incomplete covariate data. B, unstandardized regression coefficient; CI, confidence interval; SNAP, sensory nerve action potential.
